# LY6 gene family presents a novel class of potential biomarkers associated with overall survival outcome of pancreatic ductal adenocarcinoma

**DOI:** 10.18632/oncotarget.27968

**Published:** 2021-06-08

**Authors:** Claudia Gravekamp

**Keywords:** LY6 gene family, pancreatic ductal adenocarcinoma, bio marker, immune cell infiltrate

***News on:** High mRNA expression of LY6 gene family is associated with overall survival outcome in pancreatic ductal adenocarcinoma by Russ et al. Oncotarget. 2021; 12:145–159. https://doi.org/10.18632/oncotarget.27880. [PubMed]*

In this issue of Oncotarget, Russ et al. presents bioinformatic analysis of LY6 gene family in clinical samples of pancreatic ductal adenocarcinoma from TCGA collection. This data analysis was made possible by the freely available big data analysis tools such as KM plotter, Cbioportal, Oncomine, USC xenabrowser. These data indicate that expression status of LY6 gene family is relevant to survival outcome of pancreatic cancer patients. Authors make an argument that it is well worth to further investigate the casual role of LY6 gene family in therapeutic response and progression of pancreatic ductal carcinoma.

The data indicate that patient tumor samples with increased expression of LY6 genes were most relevant for cases with increased mesenchymal stem cells (MSCs) in their tumors. Generally, bone marrow residing MSCs, which are multipotent stem cells, are involved in repairing skeletal tissues—namely cartilage, bone, and fat [1]. The role of MSCs in pancreatic cancer microenvironment is beginning to emerge. MSC have immunomodulatory—specifically immunosuppressive properties. They can suppress T-cell proliferation, through the production of immune suppressive cytokines IL6 and IL10, or through IDO, PGE21. Although MSC also has been described to have anticancer properties [2], the study presented here clearly show that MSCs in the presence of increased expression of LY6 genes are associated with tumor promoting properties. It will be interesting to analyze if LY6 family proteins affect the function of MSCs in tumor microenvironment or whether LY6 gene expression is affected by MSCs.

The authors investigated the association of increased LY6 gene mRNA expression in tumors enriched for CD8 positive T-cells, CD4 positive T-cells, NKT cells, T-regulatory cells, macrophages and B-cells. TCGA data analysis showed a correlation between LY6 gene expression levels, tumor samples enriched for various immune cell infiltrate, and patient’s survival. While many LY6 genes showed increased expression associated with poor outcome to pancreatic cancer independent of their immune cell presence in tumor microenvironment, some LY6 genes were associated with OS outcome based on the status of immune cells. Increased expression of LYPD2 and PLUAR were associated with poor OS in T-reg enriched patient population, while high mRNA of CD59 and LY6G6F were associated with poor OS in T-reg decreased patient population. High mRNA of LYPD2, PLAUR and LY6E were associated with poor OS in CD8 positive T-cells enriched patient population, while high expression of CD59, LY6G6C, LY6G6D, and LYPD4 was associated with poor OS outcome only in CD8 decreased population. The interplay of LY6 genes in pancreatic tumors with immune infiltration is interesting. Some of the LY6 genes were consistently shown to be of good outcome in pancreatic cancer patients. Future research is needed to see what underlines for the effect on poor vs good clinical outcome.

Based on the findings in the Russ et al. study and the fact that LY6 genes are cell surface proteins [3], they are very well-suited for future therapeutic development. It is clear that LY6 genes are involved in many aspects of pancreatic cancer outcome. It will be interesting to determine which cell types in the tumor microenvironment express LY6, and the mechanistic role of LY6 gene family protein in pancreatic cancer and its effect on the tumor microenvironment. The Russ et al. study showed association of LY6 and immune suppression in the tumor microenvironment. Targeting LY6 by immunotherapy not only may destroy LY6-expressing cells resulting in decreased tumor progression, but simultaneously reduce immune suppression in the tumor microenvironment resulting in improved T-cell responses against the tumor. Cancer stem cells play an important role in tumor cell dissociation and the exponential development of metastases, particularly in pancreatic cancer [4]. Therefore, it is of clinical relevance that the Russ et al. study found association between cancer stem cell maintenance and high expression of the LY6 family. In summary, it is important to evaluate what drives the increased gene expression of LY6 gene family in pancreatic cancer. More insight in this field may lead to more effective treatments. Nonetheless, these clinical associations presented by Russ et al. presents us with a set of clinically relevant genes to further advance the development of novel therapeutic targets in pancreatic cancer.
